# Wealth Distribution Under Power Trading Frequencies and Transitions of Agents

**DOI:** 10.3390/e27121209

**Published:** 2025-11-28

**Authors:** Rongmei Sun, Shaoyong Lai, Xia Zhou

**Affiliations:** 1 School of Mathematics, Southwestern University of Finance and Economics, Chengdu 611130, China; laishaoy@swufe.edu.cn; 2 School of Mathematics and Big Data, Mianyang Teachers’ College, Mianyang 621000, China; xiazhou2017@163.com

**Keywords:** kinetic model, power collision kernels, bimodal distribution, Boltzmann equation, Fokker–Planck equation

## Abstract

We construct a kinetic model to investigate transactions among two populations from different countries. In our model, power collision kernels and a transfer operator are introduced into the Boltzmann equation. Through the method of continuous trading limits, we derive the Fokker–Planck equations and find their entropy steady-state solutions for two special cases. Numerical experiments are provided to illustrate our results under different parameters. The conclusion shows that the increase of agents’ trading willingness and the decrease of agents’ transaction rate (the increase of saving rate) narrow the gap in agents’ wealth. If there is a significant disparity in the trading willingness between agents from the two countries, the overall wealth distribution exhibits a bimodal distribution pattern.

## 1. Introduction

The kinetic theory of rarefied gas is one of the important components of theoretical physics, which is proposed by the works in Maxwell [1] and Boltzmann [2]. In [1], Maxwell derives the distribution function of the velocity of the rarefied gas in a steady state. Consequently, Boltzmann [2] establishes an integral differential equation to describe the time evolution of the density distribution of rarefied gas molecules, which is called the Boltzmann equation and is expressed as

$$\frac{\partial f(x,v,t)}{\partial t}=-v\cdot \nabla_{x}f\left(x,v,t\right)+Q\left(f,f\right)\left(x,v,t\right),$$

where f(x,v,t) represents the probability density function of gas molecules at time *t*, which is uniquely characterized by the position *x* of the molecule and the velocity *v* of the molecule’s motion. On the right side of the Boltzmann equation, $-v\cdot \nabla_{x}f\left(x,v,t\right)$ describes the effect of the movement of the gas molecules on the evolution of the distribution function, and Q(f,f)(x,v,t) depicts the effect of collisions between the gas molecules on the evolution of the distribution function. Subsequently, the Boltzmann equation is widely used in the field of physics. However, it is difficult to solve the Boltzmann equation in general (see [3,4,5]). A few decades ago, scholars began applying the Boltzmann equation to investigate various problems, including the distributions of traffic velocity (see [6,7,8]), cell mutation rate (see [9,10]), opinion formation (see [11,12,13,14,15]) and call center service time (see [16,17,18]).

The kinetic theory of rarefied gas has also been applied in the field of economics, where wealth distribution in the multi-agent system is one of the hot topics. In 1897, Pareto [19] found that the wealth distributions in Western countries follow Pareto’s law, which became the basis of subsequent research on wealth distribution. Pareto’s law uses the Pareto index to measure the degree of inequality of wealth distribution in a society. Utilizing *w* to denote the wealth of agents, if the steady-state wealth distribution follows the form

$$f_{\infty }\left(w\right)∼w^{-(1+\gamma )},$$

we call γ the Pareto index [20,21]. When the Pareto index γ is close to 1, most of the wealth in the society is owned by a small percentage of people, that is, wealth is distributed unevenly. Cordier et al. [22] introduce the saving parameter into the interaction rule, establish the Boltzmann equation of wealth distribution, derive the corresponding Fokker–Planck equation, and obtain its steady-state wealth distribution, which has a Pareto tail. The model constructed in [22] is called the CPT (Cordier–Pareschi–Toscani) model. Based on the interaction rule in [22], Düring and Toscani [23] modify the saving parameters so that different groups have different saving parameters. The conclusion in [23] shows that when there is a large gap between the saving parameters of the two groups of agents, the social total wealth distribution curve presents a bimodal pattern. Bisi et al. [24] introduce the mechanism of taxation and redistribution into the dynamic model and find that taxation and redistribution are capable of narrowing the wealth gap. Pareschi and Toscani [25] add the knowledge variable to the interaction rule, acquire the agent’s joint distribution of knowledge and wealth, and obtain a conclusion that knowledge widens the gap in wealth distribution. Assuming that the saving parameter in the interaction rule depends on the wealth level of the agent, Bisi [26] proves that the Pareto index of wealth distribution is affected by the trading strategies of agents. Dimarco et al. [27] combine the dynamic models of epidemiology and wealth exchange, establish Boltzmann equations to describe the evolution processes of wealth densities in susceptible, infected and recovered groups, and confirm that the spread of disease affects the wealth distribution. On the basis of the research of Dimarco et al. [27], Zhang et al. [28] derive the specific wealth distributions of both the susceptible and infected populations, which are found to follow unimodal inverse gamma distributions. Assuming that the two groups of agents have different trading propensities for the two types of goods, Sun and Wang [29] derive interaction rules based on the utility principle, finding that agents’ differing trading propensities for two goods affect wealth distribution.

In the Boltzmann equation, the collision kernel determines the interaction frequency between agents. The collision kernels are categorized into Maxwellian collision kernels (constant collision kernels) and non-Maxwellian collision kernels. The advantage of the Maxwellian collision kernel lies in its computational simplicity, but its limitation is that it fails to exclude agents with zero wealth from transactions, which does not align with real trading practices. In order to compensate for this deficiency, non-Maxwellian collision kernels are used in dynamic models to investigate the economic and social issues. Furioli et al. [30] introduce a non-Maxwellian collision kernel into the dynamic model to make the trading frequency depend on the wealth levels of both sides in the transaction and prove that the solution of the Fokker–Planck equation converges exponentially to an equilibrium value. Ballante et al. [31] use the power collision kernel and improve the interaction rule to make the agent’s saving propensity be affected by random factors. The conclusion in [31] states that under certain conditions, the social total wealth distribution curve presents a bimodal pattern. Dimarco and Toscani [32] establish a Boltzmann equation containing the power collision kernel to study the social climbing problem and find that the steady-state distribution of social status is close to Amoroso distribution. Wang and Lai [33] construct a kinetic model to capture the evolutionary patterns of wealth distribution in the financial market, and they incorporate a wealth-dependent collision kernel to investigate the impact of varying trading frequencies on wealth distribution. Assuming that both the trading frequency and trading propensity depend on the agents’ wealth, Liu et al. [34] conclude that when the trading propensity is an increasing function of wealth, the rich invest a large proportion of their wealth in transactions, leading to a reduction in the wealth gap among agents.

Assuming that the transaction frequency is a constant, Bisi [35] establishes Boltzmann equations with individual transfer to describe the wealth distribution dynamics of agents in two countries (countries 1 and 2) and obtains a steady-state wealth distribution with a Pareto tail. Our study differs from that in Bisi [35] in the following aspects.

(i) Inspired by the work in Furioli et al. [30], we choose the power collision kernels $K_{1}\left(w\right)=kw^{\alpha }$ and $K_{2}\left(w\right)=kw^{\beta }$ (*w* indicates the wealth of agents, α and β represent the intensities of trading willingness of the agents in counties 1 and 2, respectively) that differ from the constant collision kernel in Bisi [35]. The power collision kernels ensure that the agent with zero wealth does not participate in the transaction, and the transaction frequency depends on the agent’s wealth level and the trading willingness.
(ii) The conclusion in Bisi [35] illustrates that the steady-state wealth distribution contains a Pareto tail, which depends on the trading rate. However, in our steady-state solution, the Pareto index is jointly determined by the trading rate and the intensity of trading willingness.
(iii) Assuming that there is only one group in the market when t→∞, Bisi [35] obtains the wealth distribution of agents in this group, which is a unimodal distribution. In this paper, we follow the idea in Zhang et al. [28] and suppose that the distribution functions of agents in two countries are linearly correlated as t→∞ and find the steady-state distribution of the total wealth, which shows a bimodal pattern.

This paper is organized as follows. In Section 2, we give the relevant definitions of wealth distribution and the interaction rule. In Section 3, we discuss the Boltzmann equation and present the binary interaction operator and the agent transition operator. The objective of Section 4 is to deduce the Fokker–Planck equation and find its steady-state solution for two special cases. Section 5 provides the results of numerical experiments. The conclusion is presented in Section 6.

## 2. Wealth Dynamics in International Trade

In this paper, we assume that there are two countries in the market, and agents from these two countries are free to conduct domestic and international trade. Domestic trade means that both parties in the transaction are from country 1 or country 2. International trade means that one party is from country 1 and another party is from country 2. Based on this, we build a dynamic model of international trade that allows agents to change their nationalities. Transactions between agents are modeled by the binary interaction rule, and the transfers of agents (changes of agents’ nationalities) are described by Boltzmann operators.

We aim at analyzing the evolution of wealth distributions of agents in two countries. We use $f_{i}\left(w,t\right)$ (i=1,2) to denote the wealth density function of the agent in country *i*, which depends on the level of personal wealth w>0 and time t>0. As usual, in such kinetic models of wealth distribution, we do not allow agents to be in debt, that is, no agent has a negative wealth level. To maintain the continuity of wealth density function $f_{i}\left(w,t\right)$ at w→0, we set $f_{i}\left(0,t\right)=0$. The total wealth distribution is written as

$$f\left(w,t\right)=f_{1}\left(w,t\right)+f_{2}\left(w,t\right).$$

The total wealth distribution f(w,t) satisfies

$$\int_{R_{+}}f\left(w,t\right)dw=1,\;t>0.$$

The moments of the wealth distribution $f_{i}\left(w,t\right)$ are defined as

$$m_{i,s}\left(t\right)=\int_{R_{+}}w^{s}f_{i}\left(w,t\right)dw,\;s\geq 0,$$

where *s* represents the order of moments. The option of s=0 indicates the proportion of agents in each country to the total number of agents in the market

$$\rho_{1}\left(t\right)=\int_{R_{+}}f_{1}\left(w,t\right)dw,\;\rho_{2}\left(t\right)=\int_{R_{+}}f_{2}\left(w,t\right)dw.$$

$\rho_{1}\left(t\right)$ and $\rho_{2}\left(t\right)$ change with the transfers of agents. According to the property of the probability density function f(w,t), we obtain

$$\rho_{1}\left(t\right)+\rho_{2}\left(t\right)=1,\;t>0.$$

The option s=1 denotes the mean wealth of agents in each country:

$$m_{1,1}\left(t\right)=\int_{R_{+}}wf_{1}\left(w,t\right)dw,\;m_{2,1}\left(t\right)=\int_{R_{+}}wf_{2}\left(w,t\right)dw.$$

When t→∞, we assume that ${\left(m_{1,1}\right)}_{\infty }$ and ${\left(m_{2,1}\right)}_{\infty }$ satisfy

$${\left(m_{1,1}\right)}_{\infty }+{\left(m_{2,1}\right)}_{\infty }=\int_{R_{+}}wf_{\infty }\left(w\right)dw=\bar{m},$$

where $\bar{m}$ is a constant.

The distribution function $f_{i}\left(w,t\right)$ satisfies the evolution equation

(1) $$\frac{\partial f_{i}\left(w,t\right)}{\partial t}=\sum_{j=1}^{2}Q_{i}\left(f_{i},f_{j}\right)\left(w\right)+Q_{i}^{T}\left(f_{1},f_{2}\right)\left(w\right),\;i=1,2,$$

where the binary interaction operator $Q_{i}\left(f_{i},f_{j}\right)\left(w\right)$ describes the effects of binary trading between agents on the evolution of wealth distribution, and the transfer operator $Q_{i}^{T}\left(f_{1},f_{2}\right)\left(w\right)$ describes the influence of the immigration behaviors of agents on the evolution of wealth distribution.

We consider the wealth interaction in Equation (1), using (w,v) and $\left(w^{*},v^{*}\right)$ to represent the wealth of both parties before and after the transaction, respectively. The binary transactions are characterized by

(2) $$\left\{\begin{array}{c} w^{*}=w-\theta w+\theta v+\eta_{1}w,\\ v^{*}=v-\theta v+\theta w+\eta_{2}v, \end{array}\right.$$

where θ represents the trading rate of the agent. Agents having a trading rate of θ are willing to allocate a proportion θ of their total wealth to transactions, and the remaining proportion (1−θ) to savings. $\eta_{1}$ and $\eta_{2}$ are independent identically distributed random variables. In market transactions, random variables characterize the impact of certain random factors, such as unexpected events, liquidity shocks, and the collective psychological state of investors. $\eta_{1}$ and $\eta_{2}$ satisfy $\langle \eta_{1}\rangle =\langle \eta_{2}\rangle =0$ (〈·〉 denotes the mathematical expectation) and $Var\left(\eta_{1}\right)=Var\left(\eta_{2}\right)=\sigma$.

## 3. Boltzmann Equations with Interaction and Transfer Operators

In this section, we construct a dynamical model for international trade between agents from two countries. Firstly, we define the binary interaction operator $Q_{i}\left(f_{i},f_{j}\right)\left(w\right)\left(i,j=1,2\right)$ in Equation (1). Since there are random variables $\eta_{1}$ and $\eta_{2}$ in the exchange rule (2), the interaction operator is analyzed in terms of its mean value, that is,

(3) $$\int_{R_{+}}\varphi \left(w\right)Q_{i}\left(f_{i},f_{j}\right)\left(w\right)dw=\langle \int_{R_{+}^{2}}K_{i}\left(w\right)\left[\varphi \left(w^{*}\right)-\varphi \left(w\right)\right]f_{i}\left(w\right)f_{j}\left(v\right)dwdv\rangle ,$$

where 〈·〉 denotes the mathematical expectation, and φ(w) is a smooth function with supported set in $R_{+}$. In Equation (3), the frequency of binary interaction is characterized by the collision kernel $K_{i}\left(w\right)\left(i=1,2\right)$. We introduce a class of power collision kernels which makes the frequency of transaction depend on the wealth and trading willingness of the agent in country *i* and excludes agents with zero wealth from the transaction. Since there are agents from two countries in the market, the collision kernels have two specific forms, α and β, to represent the intensities of trading willingness of angents from countries 1 and 2, respectively. The collision kernels of the agents from two countries are defined as

$$K_{1}\left(w\right)=kw^{\alpha },\;K_{2}\left(w\right)=kw^{\beta }.$$

Large α and β values correspond to a high trading frequency of agents, that is to say, agents have a strong willingness to trade.

Then, we construct the transfer operator $Q_{i}^{T}\left(f_{1},f_{2}\right)\left(w\right)$ to describe the effect of agents’ immigration behaviors on wealth distribution. We consider situations where one of the parties in the transaction changes nationality, including the following four cases

$$\begin{array}{cc} \; & (a)1+1\to 1+2,\;(b)2+2\to 1+2,\;(c)1+2\to 1+1,\;(d)1+2\to 2+2. \end{array}$$

Supposing that the probability of each agent emigrating to country 2 from 1 (transfers (a) and (d)) is $\lambda_{12}$, and the probability of each agent emigrating to country 1 from 2 (transfers (b) and (c)) is $\lambda_{21}$. The total transfer operator is written as the sum of four types of transfer operators

$$Q_{i}^{T}\left(f_{1},f_{2}\right)\left(w\right)=\sum_{l\in \{a,b,c,d\}}Q_{i}^{T(l)}\left(f_{1},f_{2}\right)\left(w\right).$$

In transfers (a)–(d), agents migrate from one country to another, and the exchange of wealth generated by this process is small. We acquire the weak forms of the transfer operators $Q_{i}^{T(l)}\left(f_{1},f_{2}\right)\left(w\right)\left(l\in \left\{a,b,c,d\right\},i=1,2\right)$

$$\begin{array}{cc} \int_{R_{+}}\varphi \left(w\right)Q_{1}^{T(a)}\left(w\right)dw= & \lambda_{12}\int_{R_{+}^{2}}\varphi \left(w\right)f_{1}\left(w_{*}\right)f_{1}\left(v_{*}\right)dw_{*}dv_{*}-2\lambda_{12}\int_{R_{+}^{2}}\varphi \left(w\right)f_{1}\left(w\right)f_{1}\left(v\right)dwdv, \end{array}$$

$$\begin{array}{c} \int_{R_{+}}\varphi \left(w\right)Q_{2}^{T(a)}\left(w\right)dw=\lambda_{12}\int_{R_{+}^{2}}\varphi \left(w\right)f_{1}\left(w_{*}\right)f_{1}\left(v_{*}\right)dw_{*}dv_{*}, \end{array}$$

$$\begin{array}{c} \int_{R_{+}}\varphi \left(w\right)Q_{1}^{T(b)}\left(w\right)dw=\lambda_{21}\int_{R_{+}^{2}}\varphi \left(w\right)f_{2}\left(w_{*}\right)f_{2}\left(v_{*}\right)dw_{*}dv_{*}, \end{array}$$

$$\begin{array}{cc} \int_{R_{+}}\varphi \left(w\right)Q_{2}^{T(b)}\left(w\right)dw= & \lambda_{21}\int_{R_{+}^{2}}\varphi \left(w\right)f_{2}\left(w_{*}\right)f_{2}\left(v_{*}\right)dw_{*}dv_{*}-2\lambda_{21}\int_{R_{+}^{2}}\varphi \left(w\right)f_{2}\left(w\right)f_{2}\left(v\right)dwdv, \end{array}$$

$$\begin{array}{cc} \int_{R_{+}}\varphi \left(w\right)Q_{1}^{T(c)}\left(w\right)dw= & \lambda_{21}\int_{R_{+}^{2}}\varphi \left(w\right)f_{1}\left(w_{*}\right)f_{2}\left(v_{*}\right)dw_{*}dv_{*}+\lambda_{21}\int_{R_{+}^{2}}\varphi \left(w\right)f_{1}\left({\bar{w}}_{*}\right)f_{2}\left({\bar{v}}_{*}\right)d{\bar{w}}_{*}d{\bar{v}}_{*}\\ & -\lambda_{21}\int_{R_{+}^{2}}\varphi \left(w\right)f_{1}\left(w\right)f_{2}\left(v\right)dwdv, \end{array}$$

$$\begin{array}{c} \int_{R_{+}}\varphi \left(w\right)Q_{2}^{T(c)}\left(w\right)dw=\lambda_{21}\int_{R_{+}^{2}}\varphi \left(w\right)f_{2}\left(w\right)f_{1}\left(v\right)dwdv, \end{array}$$

$$\begin{array}{c} \int_{R_{+}}\varphi \left(w\right)Q_{1}^{T(d)}\left(w\right)dw=-\lambda_{12}\int_{R_{+}^{2}}\varphi \left(w\right)f_{1}\left(w\right)f_{2}\left(v\right)dwdv, \end{array}$$

$$\begin{array}{cc} \int_{R_{+}}\varphi \left(w\right)Q_{2}^{T(d)}\left(w\right)dw= & \lambda_{12}\int_{R_{+}^{2}}\varphi \left(w\right)f_{1}\left(w_{*}\right)f_{2}\left(v_{*}\right)dw_{*}dv_{*}+\lambda_{12}\int_{R_{+}^{2}}\varphi \left(w\right)f_{1}\left({\bar{w}}_{*}\right)f_{2}\left({\bar{v}}_{*}\right)d{\bar{w}}_{*}d{\bar{v}}_{*}\\ & -\lambda_{12}\int_{R_{+}^{2}}\varphi \left(w\right)f_{1}\left(v\right)f_{2}\left(w\right)dwdv, \end{array}$$

where $\left(w_{*},v_{*}\right)$ and $\left({\bar{w}}_{*},{\bar{v}}_{*}\right)$ represent the wealth of agents before the transition, and the wealth of agents after the transition is (w,v). In transfers (c) and (d), the two parties are from different countries; thus, it is unreasonable to consider them as homogeneous agents. Therefore, it is worth noting that $\left({\bar{w}}_{*},{\bar{v}}_{*}\right)\neq \left(v_{*},w_{*}\right)$. We write Equation (1) in weak form to acquire the following system

(4) $$\begin{array}{cc} \; & \frac{d}{dt}\int_{R_{+}}\varphi \left(w\right)f_{1}\left(w,t\right)dw\\ & =\int_{R_{+}^{2}}K_{1}\left(w\right)\langle \varphi \left(w^{*}\right)-\varphi \left(w\right)\rangle f_{1}\left(w\right)f_{1}\left(v\right)dwdv+\int_{R_{+}^{2}}K_{1}\left(w\right)\langle \varphi \left(w^{*}\right)-\varphi \left(w\right)\rangle f_{1}\left(w\right)f_{2}\left(v\right)dwdv\\ & \;+\lambda_{12}\int_{R_{+}^{2}}\varphi \left(w\right)f_{1}\left(w_{*}\right)f_{1}\left(v_{*}\right)dw_{*}dv_{*}-2\lambda_{12}\int_{R_{+}^{2}}\varphi \left(w\right)f_{1}\left(w\right)f_{1}\left(v\right)dwdv\\ & \;+\lambda_{21}\int_{R_{+}^{2}}\varphi \left(w\right)f_{2}\left(w_{*}\right)f_{2}\left(v_{*}\right)dw_{*}dv_{*}+\lambda_{21}\int_{R_{+}^{2}}\varphi \left(w\right)f_{2}\left(w_{*}\right)f_{1}\left(v_{*}\right)dw_{*}dv_{*}\\ & \;+\lambda_{21}\int_{R_{+}^{2}}\varphi \left(w\right)f_{2}\left({\bar{w}}_{*}\right)f_{1}\left({\bar{v}}_{*}\right)d{\bar{w}}_{*}d{\bar{v}}_{*}-\lambda_{21}\int_{R_{+}^{2}}\varphi \left(w\right)f_{2}\left(w\right)f_{1}\left(v\right)dwdv\\ & \;-\lambda_{12}\int_{R_{+}^{2}}\varphi \left(w\right)f_{1}\left(w\right)f_{2}\left(v\right)dwdv \end{array}$$

and

(5) $$\begin{array}{cc} \; & \frac{d}{dt}\int_{R_{+}}\varphi \left(w\right)f_{2}\left(w,t\right)dw\\ & =\int_{R_{+}^{2}}K_{2}\left(w\right)\langle \varphi \left(w^{*}\right)-\varphi \left(w\right)\rangle f_{2}\left(w\right)f_{2}\left(v\right)dwdv+\int_{R_{+}^{2}}K_{2}\left(w\right)\langle \varphi \left(w^{*}\right)-\varphi \left(w\right)\rangle f_{2}\left(w\right)f_{1}\left(v\right)dwdv\\ & \;+\lambda_{12}\int_{R_{+}^{2}}\varphi \left(w\right)f_{1}\left(w_{*}\right)f_{1}\left(v_{*}\right)dw_{*}dv_{*}+\lambda_{21}\int_{R_{+}^{2}}\varphi \left(w\right)f_{2}\left(w_{*}\right)f_{2}\left(v_{*}\right)dw_{*}dv_{*}\\ & \;-2\lambda_{21}\int_{R_{+}^{2}}\varphi \left(w\right)f_{2}\left(w\right)f_{2}\left(v\right)dwdv+\lambda_{21}\int_{R_{+}^{2}}\varphi \left(w\right)f_{2}\left(w\right)f_{1}\left(v\right)dwdv\\ & \;+\lambda_{12}\int_{R_{+}^{2}}\varphi \left(w\right)f_{1}\left(w_{*}\right)f_{2}\left(v_{*}\right)dw_{*}dv_{*}+\lambda_{12}\int_{R_{+}^{2}}\varphi \left(w\right)f_{1}\left({\bar{w}}_{*}\right)f_{2}\left({\bar{v}}_{*}\right)d{\bar{w}}_{*}d{\bar{v}}_{*}\\ & \;-\lambda_{12}\int_{R_{+}^{2}}\varphi \left(w\right)f_{1}\left(w\right)f_{2}\left(v\right)dwdv. \end{array}$$

The main notations introduced in this paper are summarized in the Table 1.

## 4. Continuous Trading Limit and Fokker–Planck Equations

According to the ideas in Bisi [35], it is difficult to deduce the analytic properties of the steady-state distribution function directly from the Boltzmann equation. We employ the “continuous trading limit” method to transform the Boltzmann equations into the Fokker–Planck equations, and we obtain the steady-state solution of the wealth distribution under certain conditions. Under appropriate asymptotic limits, the solution of the Boltzmann equation will converge to the solution of the Fokker–Planck equation [3,22]. A constant 0<ε≪1 is used to scale the parameters, and the entire market is regarded as a continuous market composing of infinite tiny transactions. The scaled parameters are as follows.

$$\theta \to \varepsilon \theta ,\;\lambda_{12}\to \varepsilon \lambda_{12},\;\lambda_{21}\to \varepsilon \lambda_{21},\;\eta_{1}\to \sqrt{\varepsilon }\eta_{1},\;\eta_{2}\to \sqrt{\varepsilon }\eta_{2}.$$

According to the interaction rule (2), we obtain

(6) $$\langle w^{*}-w\rangle =\varepsilon \theta \left(v-w\right),\;\langle {\left(w^{*}-w\right)}^{2}\rangle =\varepsilon^{2}\theta^{2}{\left(v-w\right)}^{2}+\varepsilon \sigma w^{2}.$$

Expanding the smooth function $\varphi (w^{*})$ at *w* to the second order by using Taylor’s formula, we acquire

(7) $$\varphi \left(w^{*}\right)-\varphi \left(w\right)=\varphi^{\prime }\left(w\right)\left(w^{*}-w\right)+\frac{\varphi^{\prime \prime }\left(w\right)}{2}{\left(w^{*}-w\right)}^{2}+\frac{\varphi^{\prime \prime \prime }\left(\tilde{w}\right)}{6}{\left(w^{*}-w\right)}^{3},$$

where $\tilde{w}=\xi w^{*}+\left(1-\xi \right)w$(0≤ξ≤1). Substituting formula (6) into (7), we have

(8) $$\langle \varphi \left(w^{*}\right)-\varphi \left(w\right)\rangle =\varepsilon \theta \varphi^{\prime }\left(w\right)\left(v-w\right)+\frac{\varepsilon \sigma }{2}\varphi^{\prime \prime }\left(w\right)w^{2}+R_{\varepsilon }\left(w,v\right),$$

where $R_{\varepsilon }\left(w,v\right)=\frac{\varepsilon^{2}\theta^{2}}{2}\varphi^{\prime \prime }\left(w\right){\left(v-w\right)}^{2}+\langle \frac{\varphi^{\prime \prime \prime }\left(\tilde{w}\right)}{6}{\left(w^{*}-w\right)}^{3}\rangle$. Considering Equations (4) and (8), the first and second terms in Equation (4) become

(9) $$\begin{array}{cc} \; & \int_{R_{+}^{2}}K_{1}\left(w\right)\langle \varphi \left(w^{*}\right)-\varphi \left(w\right)\rangle f_{1}\left(w\right)f_{1}\left(v\right)dwdv\\ & \;+\int_{R_{+}^{2}}K_{1}\left(w\right)\langle \varphi \left(w^{*}\right)-\varphi \left(w\right)\rangle f_{1}\left(w\right)f_{2}\left(v\right)dwdv\\ & =\int_{R_{+}^{2}}\varepsilon k\theta w^{\alpha }\left[\varphi^{\prime }\left(w\right)\left(v-w\right)+\frac{\sigma }{2}\varphi^{\prime \prime }\left(w\right)w^{2}\right]f_{1}\left(w\right)f_{1}\left(v\right)dwdv\\ & \;+\int_{R_{+}^{2}}\varepsilon k\theta w^{\alpha }\left[\varphi^{\prime }\left(w\right)\left(v-w\right)+\frac{\sigma }{2}\varphi^{\prime \prime }\left(w\right)w^{2}\right]f_{1}\left(w\right)f_{2}\left(v\right)dwdv+{\tilde{R}}_{\varepsilon }\left(w,v\right), \end{array}$$

where

$$\begin{array}{cc} {\tilde{R}}_{\varepsilon }\left(w,v\right)= & k[\int_{R_{+}^{2}}w^{\alpha }R_{\varepsilon }\left(w,v\right)f_{1}\left(w\right)f_{1}\left(v\right)dwdv\\ \; & +\int_{R_{+}^{2}}w^{\alpha }R_{\varepsilon }\left(w,v\right)f_{1}\left(w\right)f_{2}\left(v\right)dwdv]. \end{array}$$

Then, we scale the time τ=εt so that the scaled wealth distribution functions satisfy $g_{1}\left(w,\tau \right)=f_{1}\left(w,t\right)$ and $g_{2}\left(v,\tau \right)=f_{2}\left(v,t\right)$. When ε→0, the remainder ${\tilde{R}}_{\varepsilon }\left(w,v\right)\to 0$. Performing the integration by parts, Equation (9) becomes

(10) $$\begin{array}{cc} \; & \int_{R_{+}^{2}}K_{1}\left(w\right)\langle \varphi \left(w^{*}\right)-\varphi \left(w\right)\rangle f_{1}\left(w\right)f_{1}\left(v\right)dwdv\\ & \;+\int_{R_{+}^{2}}K_{1}\left(w\right)\langle \varphi \left(w^{*}\right)-\varphi \left(w\right)\rangle f_{1}\left(w\right)f_{2}\left(v\right)dwdv\\ \; & =-k\theta \left(m_{1,1}\left(\tau \right)+m_{2,1}\left(\tau \right)\right)\int_{R_{+}^{2}}\varphi \left(w\right)\frac{\partial }{\partial w}\left(w^{\alpha }g_{1}\left(w\right)\right)dw\\ & \;+k\theta \left(\rho_{1}\left(\tau \right)+\rho_{2}\left(\tau \right)\right)\int_{R_{+}^{2}}\varphi \left(w\right)\frac{\partial }{\partial w}\left(w^{\alpha +1}g_{1}\left(w\right)\right)dw\\ & \;+\frac{k\sigma }{2}\left(\rho_{1}\left(\tau \right)+\rho_{2}\left(\tau \right)\right)\int_{R_{+}^{2}}\varphi \left(w\right)\frac{\partial^{2}}{\partial w^{2}}\left(w^{\alpha +2}g_{1}\left(w\right)\right)dw\\ \; & =-k\theta \left(m_{1,1}\left(\tau \right)+m_{2,1}\left(\tau \right)\right)\int_{R_{+}^{2}}\varphi \left(w\right)\frac{\partial }{\partial w}\left(w^{\alpha }g_{1}\left(w\right)\right)dw\\ & \;+k\theta \int_{R_{+}^{2}}\varphi \left(w\right)\frac{\partial }{\partial w}\left(w^{\alpha +1}g_{1}\left(w\right)\right)dw\\ \; & \;+\frac{k\sigma }{2}\int_{R_{+}^{2}}\varphi \left(w\right)\frac{\partial^{2}}{\partial w^{2}}\left(w^{\alpha +2}g_{1}\left(w\right)\right)dw. \end{array}$$

We derive the Fokker–Planck equation of $g_{1}\left(w,\tau \right)$ by substituting (10) into (4)

(11) $$\begin{array}{cc} \frac{\partial g_{1}\left(w,\tau \right)}{\partial \tau }= & -k\theta \left(m_{1,1}\left(\tau \right)+m_{2,1}\left(\tau \right)\right)\frac{\partial }{\partial w}\left(w^{\alpha }g_{1}\left(w,\tau \right)\right)+k\theta \frac{\partial }{\partial w}\left(w^{\alpha +1}g_{1}\left(w,\tau \right)\right)\\ & +\frac{k\sigma }{2}\frac{\partial^{2}}{\partial w^{2}}\left(w^{\alpha +2}g_{1}\left(w,\tau \right)\right)+\lambda_{21}g_{2}\left(w,\tau \right)-\lambda_{12}g_{1}\left(w,\tau \right). \end{array}$$

Using the same method, we acquire the Fokker–Planck equation of $g_{2}\left(w,\tau \right)$

(12) $$\begin{array}{cc} \frac{\partial g_{2}\left(w,\tau \right)}{\partial \tau }= & -k\theta \left(m_{1,1}\left(\tau \right)+m_{2,1}\left(\tau \right)\right)\frac{\partial }{\partial w}\left(w^{\beta }g_{2}\left(w,\tau \right)\right)+k\theta \frac{\partial }{\partial w}\left(w^{\beta +1}g_{2}\left(w,\tau \right)\right)\\ & +\frac{k\sigma }{2}\frac{\partial^{2}}{\partial w^{2}}\left(w^{\beta +2}g_{2}\left(w,\tau \right)\right)-\lambda_{21}g_{2}\left(w,\tau \right)+\lambda_{12}g_{1}\left(w,\tau \right). \end{array}$$

The first-order partial derivative terms of wealth *w* in Equations (11) and (12) are drift terms, which make the wealth evolve toward the weighted average of wealth of the two populations. The influence of random factors leads to the spread of wealth, which is described by the diffusion terms, and the specific forms are shown in the second-order partial derivative terms in Formulas (11) and (12). Obviously, when there are no random factors in the market, the diffusion terms in the Fokker–Planck equations disappear. In general, solving Equations (11) and (12) possesses certain difficulties. Therefore, additional assumptions are required.

### 4.1. Solvable Case 1

We consider a special case where the government sets a high threshold for immigration to country 2—that is, only agents with a very high level of wealth can migrate to the country 2 from 1. At this point, the transition probability $\lambda_{12}$ becomes small and satisfies $\lambda_{12}=O\left(\varepsilon^{2}\right)$. When ε→0, we have $\lambda_{12}=0$. In this situation, the entire market reaches a steady state where all agents live in the country 1, and we have ${\left(\rho_{2}\right)}_{\infty }=0$, ${\left(m_{2,1}\right)}_{\infty }=0$, ${\left(\rho_{1}\right)}_{\infty }=1$ and ${\left(m_{1,1}\right)}_{\infty }=\bar{m}$ when t→∞. Consequently, the steady-state distribution ${\left(g_{2}\right)}_{\infty }\left(w\right)$ vanishes, and ${\left(g_{1}\right)}_{\infty }\left(w\right)$ is the solution of the following equation

(13) $$\begin{array}{c} -\theta \bar{m}\frac{\partial }{\partial w}\left(w^{\alpha }{\left(g_{1}\right)}_{\infty }\left(w\right)\right)+\theta \frac{\partial }{\partial w}\left(w^{\alpha +1}{\left(g_{1}\right)}_{\infty }\left(w\right)\right)+\frac{\sigma }{2}\frac{\partial^{2}}{\partial w^{2}}\left(w^{\alpha +2}{\left(g_{1}\right)}_{\infty }\left(w\right)\right)=0. \end{array}$$

Solving Equation (13), we obtain

(14) $${\left(g_{1}\right)}_{\infty }\left(w\right)=C_{1}w^{-\left(\frac{2\theta }{\sigma }+\alpha +2\right)}exp\left\{-\frac{2\theta \bar{m}}{\sigma }\frac{1}{w}\right\}.$$

According to $\int_{R_{+}}{\left(g_{1}\right)}_{\infty }\left(w\right)=1$$\left({\left(\rho_{1}\right)}_{\infty }=1\right)$, we can obtain the constant $C_{1}$. The complete expression of the steady solution ${\left(g_{1}\right)}_{\infty }\left(w\right)$ is as follows

(15) $${\left(g_{1}\right)}_{\infty }\left(w\right)=\frac{{\left(\frac{2\theta \bar{m}}{\sigma }\right)}^{\frac{2\theta }{\sigma }+\alpha +1}}{\Gamma \left(\frac{2\theta }{\sigma }+\alpha +1\right)}w^{-\left(\frac{2\theta }{\sigma }+\alpha +2\right)}exp\left\{-\frac{2\theta \bar{m}}{\sigma }\frac{1}{w}\right\}.$$

From the steady-state solution (15), it can be seen that the steady-state wealth distribution of the agent follows a generalized inverse gamma distribution and the Pareto index is γ=2θ/σ+α+1. This result indicates that the social wealth distribution has a heavy tail which depends on agents’ trading rates, trading willingness, and market risks. A country with a heavy-tail phenomenon, that is, a phenomenon where a small number of people hold most of the wealth, could use solution (15) to describe the wealth distribution. In Section 5, we will show how the trading rate and the intensity of trading willingness affect the steady-state wealth distribution through numerical experiments.

### 4.2. Solvable Case 2

As a discussion of another possibility, the ratio of the two groups of agents reaches a steady state as t→∞. We assume that the wealth density of agents in the two countries is linearly correlated at t→∞; that is, the expression ${\left(g_{2}\right)}_{\infty }\left(w\right)=D{\left(g_{1}\right)}_{\infty }\left(w\right)$ is satisfied where D>0 is a constant. From an economic perspective, when the economic structures, development stages and policy directions of two countries converge in the long run, their wealth distributions may tend to change proportionally, i.e., ${\left(g_{2}\right)}_{\infty }\left(w\right)=D{\left(g_{1}\right)}_{\infty }\left(w\right)$. For instance, if both countries are dominated by high-tech industries and the service sector, and the governments of both countries adopt policies that encourage innovation and regulate excessive incomes, the patterns of wealth creation and distribution in the two countries would be similar. Over the long term, the wealth distributions of the two countries may change proportionally. According to Equations (11) and (12), ${\left(g_{1}\right)}_{\infty }\left(w\right)$ and ${\left(g_{2}\right)}_{\infty }\left(w\right)$ are solutions of the following two equations

(16) $$\begin{array}{cc} \; & -k\theta \bar{m}\frac{\partial }{\partial w}\left(w^{\alpha }{\left(g_{1}\right)}_{\infty }\left(w\right)\right)+k\theta \frac{\partial }{\partial w}\left(w^{\alpha +1}{\left(g_{1}\right)}_{\infty }\left(w\right)\right)\\ & +\frac{k\sigma }{2}\frac{\partial^{2}}{\partial w^{2}}\left(w^{\alpha +2}{\left(g_{1}\right)}_{\infty }\left(w\right)\right)+\left(\lambda_{21}D-\lambda_{12}\right){\left(g_{1}\right)}_{\infty }\left(w\right)=0 \end{array}$$

and

(17) $$\begin{array}{cc} \; & -k\theta \bar{m}\frac{\partial }{\partial w}\left(w^{\beta }{\left(g_{2}\right)}_{\infty }\left(w\right)\right)+k\theta \frac{\partial }{\partial w}\left(w^{\beta +1}{\left(g_{2}\right)}_{\infty }\left(w\right)\right)\\ & +\frac{k\sigma }{2}\frac{\partial^{2}}{\partial w^{2}}\left(w^{\beta +2}{\left(g_{2}\right)}_{\infty }\left(w\right)\right)+\left(\frac{\lambda_{12}}{D}-\lambda_{21}\right){\left(g_{2}\right)}_{\infty }\left(w\right)=0. \end{array}$$

In order to acquire the exact solutions of Equations (16) and (17), we add an assumption that the constant $D=\lambda_{12}/\lambda_{21}$. Thus, Equations (16) and (17) are simplified to

(18) $$\begin{array}{c} -k\theta \bar{m}\frac{\partial }{\partial w}\left(w^{\alpha }{\left(g_{1}\right)}_{\infty }\left(w\right)\right)+k\theta \frac{\partial }{\partial w}\left(w^{\alpha +1}{\left(g_{1}\right)}_{\infty }\left(w\right)\right)+\frac{k\sigma }{2}\frac{\partial^{2}}{\partial w^{2}}\left(w^{\alpha +2}{\left(g_{1}\right)}_{\infty }\left(w\right)\right)=0 \end{array}$$

and

(19) $$\begin{array}{c} -k\theta \bar{m}\frac{\partial }{\partial w}\left(w^{\beta }{\left(g_{2}\right)}_{\infty }\left(w\right)\right)+k\theta \frac{\partial }{\partial w}\left(w^{\beta +1}{\left(g_{2}\right)}_{\infty }\left(w\right)\right)+\frac{k\sigma }{2}\frac{\partial^{2}}{\partial w^{2}}\left(w^{\beta +2}{\left(g_{2}\right)}_{\infty }\left(w\right)\right)=0. \end{array}$$

At this point, since there are two groups of agents in the market, ${\left(g_{1}\right)}_{\infty }\left(w\right)$ and ${\left(g_{2}\right)}_{\infty }\left(w\right)$ must satisfy the conditions $\int_{R_{+}}{\left(g_{1}\right)}_{\infty }\left(w\right)dw=\rho_{1}$ and $\int_{R_{+}}{\left(g_{2}\right)}_{\infty }\left(w\right)dw=\rho_{2}$$\left(\rho_{1}+\rho_{2}=1\right)$. To solve Equations (18) and (19), we obtain

$$\begin{array}{c} {\left(g_{1}\right)}_{\infty }\left(w\right)=\frac{\rho_{1}{\left(\frac{2\theta \bar{m}}{\sigma }\right)}^{\frac{2\theta }{\sigma }+\alpha +1}}{\Gamma \left(\frac{2\theta }{\sigma }+\alpha +1\right)}w^{-\left(\frac{2\theta }{\sigma }+\alpha +2\right)}exp\left\{-\frac{2\theta \bar{m}}{\sigma }\frac{1}{w}\right\} \end{array}$$

and

$${\left(g_{2}\right)}_{\infty }\left(w\right)=\frac{\rho_{2}{\left(\frac{2\theta \bar{m}}{\sigma }\right)}^{\frac{2\theta }{\sigma }+\beta +1}}{\Gamma \left(\frac{2\theta }{\sigma }+\beta +1\right)}w^{-\left(\frac{2\theta }{\sigma }+\beta +2\right)}exp\left\{-\frac{2\theta \bar{m}}{\sigma }\frac{1}{w}\right\}.$$

When α=β, the total wealth distribution follows the form of Formula (15). When α≠β, the total wealth distribution $g_{\infty }\left(w\right)={\left(g_{1}\right)}_{\infty }\left(w\right)+{\left(g_{2}\right)}_{\infty }\left(w\right)$, that is,

(20) $$\begin{array}{cc} g_{\infty }\left(w\right)= & \frac{\rho_{1}{\left(\frac{2\theta \bar{m}}{\sigma }\right)}^{\frac{2\theta }{\sigma }+\alpha +1}}{\Gamma \left(\frac{2\theta }{\sigma }+\alpha +1\right)}w^{-\left(\frac{2\theta }{\sigma }+\alpha +2\right)}exp\left\{-\frac{2\theta \bar{m}}{\sigma }\frac{1}{w}\right\}\\ \; & +\frac{\rho_{2}{\left(\frac{2\theta \bar{m}}{\sigma }\right)}^{\frac{2\theta }{\sigma }+\beta +1}}{\Gamma \left(\frac{2\theta }{\sigma }+\beta +1\right)}w^{-\left(\frac{2\theta }{\sigma }+\beta +2\right)}exp\left\{-\frac{2\theta \bar{m}}{\sigma }\frac{1}{w}\right\}. \end{array}$$

In this case, the distribution of total wealth presents the characteristics of bimodal distribution, which will be demonstrated by numerical experiments in Section 5.

## 5. Numerical Experiments

### 5.1. Test 1: Analysis of Influencing Factors of Steady-State Wealth Distribution

Since the steady-state wealth distribution ${\left(g_{1}\right)}_{\infty }\left(w\right)$ depends on the intensity of trading willingness α and the trading rate θ, when the values of α and θ are modified, the steady-state wealth distribution changes. Thus, we conduct numerical analysis from two aspects. Firstly, the effects of different trading willingness on the steady-state wealth distribution are analyzed. Then, we explore the evolution patterns of distribution curves under varying trading rate values.

**Figure 1:**
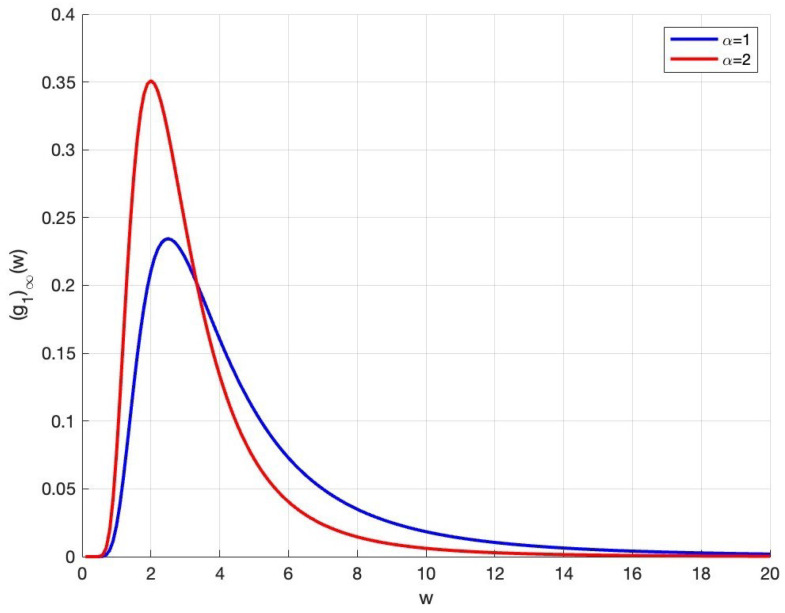
The effect of the intensity of trading willingness on steady-state wealth distribution.

Fixing parameters θ=0.5, σ=1 and $\bar{m}=10$, we know from Figure 1 that when α takes different values, the wealth distribution curves have similar trends, which rise first and then gradually decrease to 0 with the increase of wealth *w*. It is worth noting that the increase of α makes the wealth density of agents appear in a thin tail form, indicating that the increase of agents’ trading willingness helps to improve the wealth inequality.

**Figure 2:**
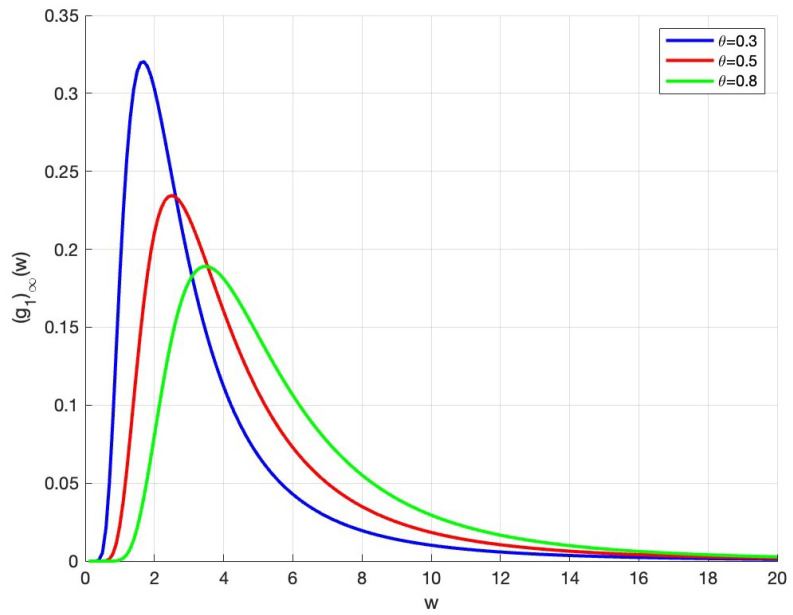
The effect of the trading rate on steady-state wealth distribution.

Moreover, setting α=1, σ=1 and $\bar{m}=10$, we visualize the wealth distribution trend when θ takes different values. As can be seen from Figure 2, when the agent’s transaction rate increases, the tail of the curve of steady-state wealth distribution thickens—that is, the wealth disparity is growing. In other words, high transaction rates reduce the wealth of the poor and increase the wealth of the rich. Therefore, the reasonable way for the poor to accumulate wealth is to improve the rate of saving.

### 5.2. Test 2: The Bimodal Characteristics of the Total Wealth Distribution

**Figure 3:**
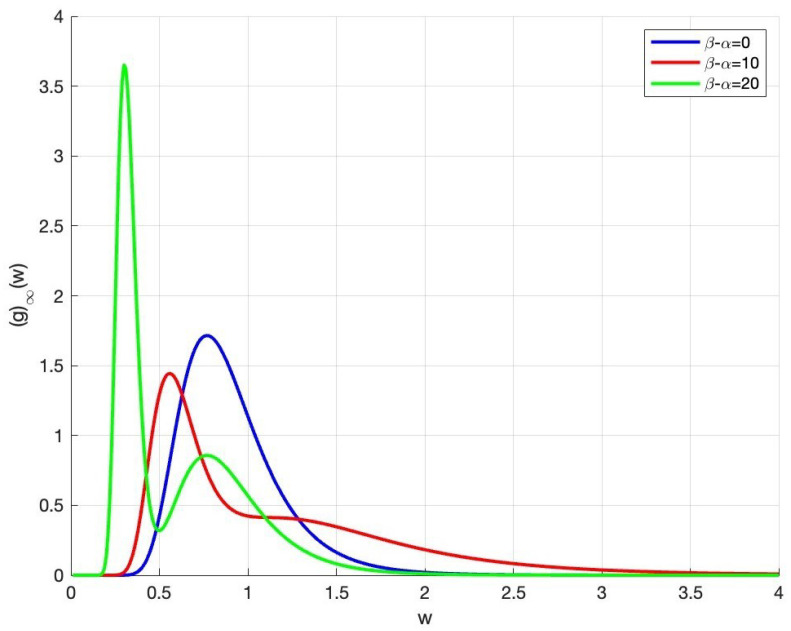
The trend graph of total wealth distribution.

We keep other parameters unchanged ($\rho_{1}=\rho_{2}=\theta =0.5,\sigma =1,\bar{m}=10$) and analyze the influence of the difference in trading willingness β−α on the total wealth distribution. In Figure 3, when the value of β−α increases, the distribution of total wealth changes from the unimodal form to the bimodal form. Moreover, the larger the value of β−α, the more obvious the bimodal pattern. The reason for the bimodal form is that for agents from two countries, the maximum values of their distribution functions correspond to different wealth levels. When the wealth distribution presents a bimodal characteristic, it usually means that two main economic groups are forming in the society, and these two groups have different wealth levels. This distribution could bring about several social impacts, such as the division of the consumer market (into high-end markets and mass markets) and the intensification of conflicts between different groups.

## 6. Final Remarks

In this paper, we utilize the kinetic theory of rarefied gas to explore the wealth distribution in international trade and introduce a power collision kernel into the kinetic model to ensure that agents with zero wealth do not participate in the transaction. The wealth distributions of both groups of agents are simultaneously influenced by binary interactions and agents’ transfer behaviors. Since it is difficult to solve the Boltzmann equations, we derive equations of the Fokker–Planck type by the continuous trading limits process and acquire the steady-state solutions for two special cases. From the specific form of the steady-state solution in solvable case 1, it can be seen that the steady-state wealth distribution of the agent has a Pareto tail, and the Pareto index γ=2θ/σ+α+1 depends on the trading rate and the intensity of trading willingness. From the steady-state distribution of total wealth in solvable case 2, we draw the conclusion that when there is a gap between the trading willingness of agents in two countries, the total wealth distribution appears to have the characteristcs of bimodal distribution. In numerical experiments, we analyze the influence of trading rate and the intensities of transaction willingness on wealth distribution. As can be seen from the visualization results, we conclude that the increase of agents’ willingness to trade leads to the reduction in wealth inequality. However, the increase in the agent’s transaction rate (the decrease of the agent’s saving rate) results in an increase in the inequality of wealth distribution. In addition, assuming that the wealth distributions of agents in two countries are linearly correlated as t→∞, we obtain the total wealth distribution, which shows a bimodal pattern when there is a big difference in the trading willingness of agents from two countries. In our future work, the two-country model could be extended to a multi-country scenario to further analyze the impact of the multi-country trade network on wealth distribution. If real transaction data of micro-individuals are disclosed, the kinetic model in this paper could be verified through empirical research. In addition, policy variables, such as tax adjustment and government transfer payments, could be introduced based on the dynamic model of this paper to explore the impact of external intervention on wealth distribution. 

## Figures and Tables

**Table 1:** Summary of key model parameters.

Parameter Symbol	Parameter Meaning	Range of Values
α	The intensity of trading willingness of agents in country 1.	0<α<+∞
β	The intensity of trading willingness of agents in country 2.	0<β<+∞
θ	The trading rate of agents.	0≤θ≤1
σ	The expectation of the square of a random variable.	0<σ<+∞
$\lambda_{12}$	The probability of an agent transferring from country 1 to country 2.	$0\leq \lambda_{12}\leq 1$
$\lambda_{21}$	The probability of an agent transferring from country 2 to country 1.	$0\leq \lambda_{21}\leq 1$
*D*	The ratio of the steady-state wealth distributions of the two countries of agents.	0<D<+∞

## Data Availability

No new data are generated or analyzed in this study; therefore, data sharing is not applicable.
